# Antiamoebic Properties of Metabolites against *Naegleria fowleri* and *Balamuthia mandrillaris*

**DOI:** 10.3390/antibiotics11050539

**Published:** 2022-04-19

**Authors:** Ruqaiyyah Siddiqui, Anania Boghossian, Bushra Khatoon, Muhammad Kawish, Ahmad M. Alharbi, Muhammad Raza Shah, Hasan Alfahemi, Naveed Ahmed Khan

**Affiliations:** 1 College of Arts and Sciences, American University of Sharjah, Sharjah 26666, United Arab Emirates; rsiddiqui@aus.edu (R.S.); anania999kb@gmail.com (A.B.); 2 H.E.J. Research Institute of Chemistry, International Centre for Chemical and Biological Sciences, University of Karachi, Karachi 75270, Pakistan; bushranaveed57@gmail.com (B.K.); kawishiqbal02@gmail.com (M.K.); raza_shahm@yahoo.com (M.R.S.); 3 Department of Clinical Laboratory Sciences, College of Applied Medical Sciences, Taif University, Taif 26521, Saudi Arabia; a.alharbii@tu.edu.sa; 4 Department of Medical Microbiology, Faculty of Medicine, Al-Baha University, Al-Baha 65731, Saudi Arabia; halfahmi@bu.edu.sa; 5 Department of Clinical Sciences, College of Medicine, University of Sharjah, Sharjah 27272, United Arab Emirates

**Keywords:** *Naegleria fowleri*, *Balamuthia mandrillaris*, *Rinorea vaundensis*, *Salvia triloba*, anti-amoebic, plant metabolites

## Abstract

*Naegleria fowleri* and *Balamuthia mandrillaris* are free-living, opportunistic protists, distributed widely in the environment. They are responsible for primary amoebic meningoencephalitis (PAM) and granulomatous amoebic encephalitis (GAE), the fatal central nervous infections with mortality rates exceeding 90%. With the rise of global warming and water shortages resulting in water storage in tanks (where these amoebae may reside), the risk of infection is increasing. Currently, as a result of a lack of awareness, many cases may be misdiagnosed. Furthermore, the high mortality rate indicates the lack of effective drugs available. In this study, secondary metabolites from the plants *Rinorea vaundensis* and *Salvia triloba* were tested for their anti-amoebic properties against *N. fowleri* and *B. mandrillaris.* Three of the nine compounds showed potent and significant anti-amoebic activities against both *N. fowleri* and *B. mandrillaris*: ursolic acid, betulinic acid, and betulin. Additionally, all compounds depicted limited or minimal toxicity to human cells and were capable of reducing amoeba-mediated host cell death. Moreover, the minimum inhibitory concentration required to inhibit 50% of amoebae growth, the half-maximal effective concentration, and the maximum non-toxic dose against human cells of the compounds were determined. These effective plant-derived compounds should be utilized as potential therapies against infections due to free-living amoebae, but future research is needed to realize these expectations.

## 1. Introduction

*Naegleria fowleri* and *Balamuthia mandrillaris* are free-living, opportunistic protists, distributed widely in the environment [1,2,3,4]. They are responsible for fatal central nervous infections, with mortality rates exceeding 90%; the infections are granulomatous amoebic encephalitis (GAE) and primary amoebic meningoencephalitis (PAM) caused by *B. mandrillaris* and *N. fowleri*, respectively [5,6,7]. Although these parasites are distributed widely in the environment, they do prefer warmer regions [3]. For this reason, with the rise of global warming, as well as the rise in outdoor activities and lack of water in developing countries, resulting in water storage in tanks (where these amoebae may reside), more awareness is needed [2,3]. Currently, many cases of infections due to these amoebae may be misdiagnosed [8,9].

It is evident from the high mortality rate, that there is a lack of effective drugs available [10,11,12]. Although many advances in antimicrobial therapy have been made, there have not been any significant advances in the development of effective drugs against brain-eating amoebae. Hence, the need to find effective therapies and drugs capable of selectively and effectively killing the parasite is essential [13,14,15]. Current treatments involve high concentrations of drugs leading to host tissue damage and toxicity, as is the case with the administration of Amphotericin B, which leads to nephrotoxicity [16,17]. The development of new drugs capable of penetrating the blood–brain barrier and targeting the pathogen are needed [18].

Plants are natural products that are rich sources of pharmacologically active agents, in fact, medicinal plants have been used by the ancient Greeks, Chinese, Indians, Romans, Egyptians, and Arabs [19,20]. Using plant-derived medicine, several pathological conditions have been treated, such as: diarrhea, constipation, and cough [19,21]. Furthermore, natural products contain a wider range of bioactive compounds compared to that of synthetic small molecules [22]. The various secondary metabolites synthesized by various plants allow them to possess antimicrobial properties, which is why the World Health Organization (WHO) refers to medicinal plants as the best source to attain a variety of drugs [23]. In this study, we hypothesize the antiamoebic properties of two medicinal plants, *Rinorea yaundensis* and *Salvia triloba*. The various secondary metabolites of these medicinal plants were isolated and characterized through phytochemical studies. The metabolites were tested against two amoebae, *N. fowleri* and *B. mandrillaris* for their antiamoebic properties, as previously we have shown that these metabolites are effective against *Acanthamoeba castellanii* [24]. The results showcased that these plant-based secondary metabolites are capable of exhibiting significant amoebicidal activities whereby displaying minimal adverse effects of toxicity against human cells. Future research is warranted to determine their translational value in the clinic to develop much-needed antimicrobials against free-living amoebae pathogens.

## 2. Materials and Methods

All chemicals were obtained from Sigma-Aldrich unless stated otherwise.

### 2.1. Collection, Identification of Plants and Metabolite Extraction

The leaves of *S. triloba* and the aerial parts of *R. yaundensis* were collected and the metabolites from the leaves of *S. triloba* and aerial parts of *R. yaundensis* were extracted as previously described [24]. The leaves of the *S. triloba* (10 Kg) and the aerial parts of *R. yaundensis* (27 Kg) were dried and extracted thrice with methanol (3 × 30 L, RT). The crude methanolic extract (405.6 g) of *S. triloba* and *R. yaundensis* were concentrated in vacuo chromatographed on silica gel with DCM, EtOAc, n-hexane, and MeOH by varying polarities. This led to the isolation of the secondary metabolites. The protocols for the isolation of both plant extracts are illustrated in [24].

### 2.2. Henrietta Lacks (HeLa) Cervical Cancer Cells

In order to maintain cultures of *B. mandrillaris* and *N. fowleri* and conduct various assays such as cytopathogenicity and cytotoxicity assays, it is necessary to grow and sustain human cells. In this study, HeLa cells were obtained from the American Type Culture Collections (ATCC), belonging to the identification ATCC CCL-2 [5]. To maintain the cells, complete media was prepared in which they were cultured. Complete media constitutes Roswell Park Memorial Media (RPMI), 10% fetal bovine serum (FBS), 1% L-glutamine, 1% minimum essential medium amino acids, and 1% penicillin-streptomycin. Additionally, the cultures were placed in 95% humidified incubators with 5% CO_2_ at 37 °C.

### 2.3. Naegleria fowleri Culture

*N. fowleri* strain (ATCC 30174) is a clinical isolate, derived from the human cerebrospinal fluid of primary amoebic meningoencephalitis case. The cultures were obtained from the American Type Culture Collection (ATCC) and cultured as described previously [5]. Briefly, the cells were maintained and cultured in RPMI. Additionally, serving as a food source, the cells were placed on HeLa cell monolayers. Furthermore, the cells were kept in a 95% humidified incubator containing 5% CO_2_ at 37 °C. After 48 h, the amoeba had consumed the HeLa cells resulting in approximately 2 × 10^5^ amoebae being present, of which 95% were in trophozoite form.

### 2.4. Balamuthia mandrillaris Culture

*B. mandrillaris* (ATCC 50209) is a clinical isolate, derived from the brain tissue of a 3-year, 10-month-old female mandrill, *Papio sphinx*, that died of amebic meningoencephalitis in San Diego Zoo. The cultures were obtained from the ATCC [5]. Briefly, the cells were maintained and cultured in RPMI. Additionally, the cells were placed on HeLa cell monolayers, serving as their food source. Furthermore, the cells were placed in a 95% humidified incubator containing 5% CO_2_ at 37 °C. After 48 h, the amoeba had consumed the HeLa cells, thus resulting in approximately 2 × 10^5^ amoebae being present.

### 2.5. Amoebicidal Assay

To evaluate the antiamoebic properties of drugs, an amoebicidal assay was conducted [5]. In a 96-well plate, 2 × 10^5^ amoebae were placed and brought to a final volume of 200 µL. The amoebae were then treated with 100 µg/mL of the plant-based drugs and incubated for 24 h at a temperature of 37 °C, with 5% CO_2_ and 95% humidity. Additionally, positive, and negative controls were set up. For the positive control, 0.25% SDS was used for the negative control, and amoeba with RPMI alone was used. The number of viable amoebae was calculated by first counting the living amoeba cells using a hemocytometer [5]. Furthermore, through the addition of 0.1% methylene blue, a distinguishment between the living and dead amoeba was made. Moreover, to determine whether the amoebicidal activity is significant or not, a Student’s *t*-test with two-tailed distribution was performed [5]. Moreover, certain compounds were tested at different concentrations (50 µg/mL, 100 µg/mL, 150 µg/mL, and 200 µg/mL) to determine their minimum inhibitory concentrations (MIC_50_) values [25].

### 2.6. Cell Viability Assay

To determine the cell viability against these drugs, a 3-(4,5-dimethylthiazol-2-yl)-2,5-diphenyl tetrazolium bromide (MTT) assay was performed. Briefly, HeLa cells were grown up to 90% confluency in a 96 well plate for 24 h at 37 °C in a 95% humidified incubator with 5% CO_2_ [24,25]. Following the 24-h incubation period, 100 µg/mL concentrations of the drugs were added to the cells and incubated overnight. Next, 10µL of freshly prepared MTT dye solution was added, proceeding with a 4-h incubation period. Following the 4-h incubation period, 100 µL of DMSO was added to dissolve the formazan crystals formed by the cells. Additionally, HeLa cells with DMSO were taken as the negative control. The absorbance was recorded at 590 nm and the viability was calculated using the following formula: % Viability = Mean OD of test sample/Mean OD of negative control ×100. Additionally, the EC_50_ (50% effective concentration) and the MNTD (maximum non-toxic dose) was determined through the conduction of MTT assays.

### 2.7. Cytopathogenicity Assay

Cytopathogenicity assays were conducted to determine the amoebae-mediated host cell death [5]. 2 × 10^5^ amoebae were challenged with 100 µg/mL of the different compounds and were incubated for 2 h at a temperature of 37 °C with 5% CO_2_ and 95% humidity. Following the incubation period, the samples were centrifuged, and the pellet was resuspended in RPMI. Next, the resuspended pellet was added to a 96-well plate containing confluent cancer cell monolayers [5]. After 24-h, the supernatant was collected and the cytotoxicity was measured using a cytotoxicity detection kit, to measure the LDH release [5]. Both negative and positive controls were included, the negative control being the cells with RPMI only and the positive control being the host cells treated with Triton-X-100 [5].

### 2.8. Statistical Analysis

All the data obtained are illustrative of the mean ± standard error of multiple independent experiments. Additionally, the statistical significance was evaluated with the use of a two-tailed distribution *t*-test [5]. Moreover, the *p*-values were determined to further examine and elaborate on the significance of the results.

## 3. Results

### 3.1. The Plant-Based Compounds Exhibited Significant Amoebicidal Activity against N. fowleri and B. mandrillaris

**Figure 1:**
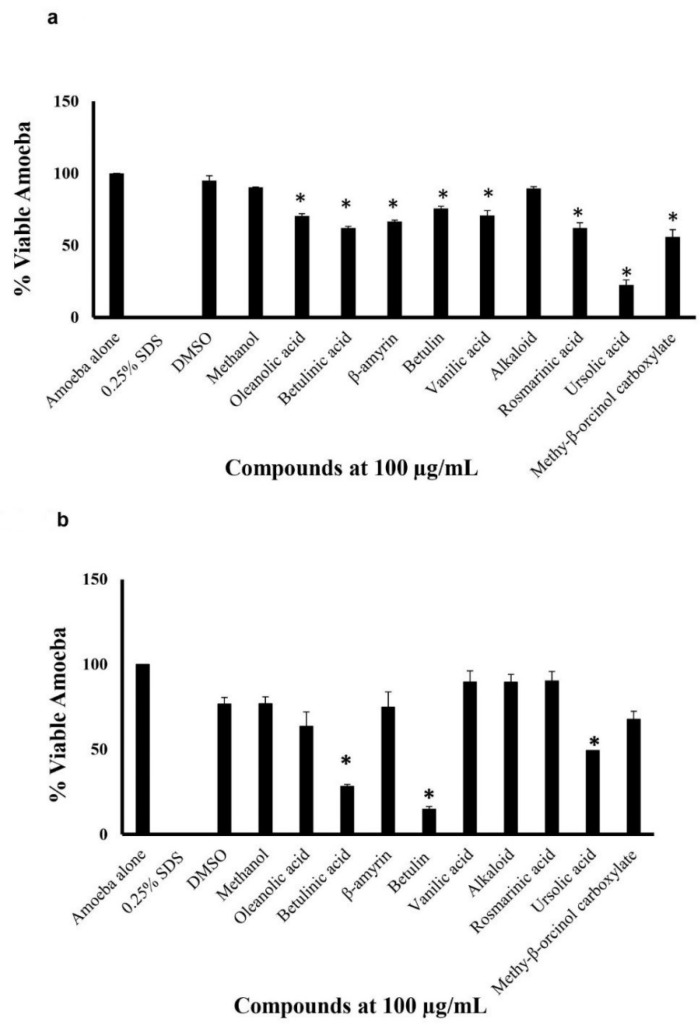
The plant-based compounds exhibited significant amoebicidal activity against *N. fowleri* and *B. mandrillaris* (**a**) effects of the drugs against B. mandrillaris after a 24-h incubation period and (**b**) effects of the drugs against *N. fowleri* after a 24-h incubation period. The data is illustrative of several independent experiments and presented as the mean ± standard error. Furthermore, *p*-values were determined using two-sample *t*-test, two-tailed distribution, (*) is ≤0.05.

To determine the antiamoebic effects of the plant-based natural compounds amoebicidal assays were performed against *N. fowleri* and *B. mandrillaris.* The results showed upon the 24-h incubation of the pathogen with 100 µg/mL of the plant-based compounds, certain compounds showed significant amoebicidal activity (*t*-test, two-tail distribution, *p* ≤ 0.05) (Figure 1a,b). All compounds, except alkaloid, showcased significant amoebicidal activity against *B. mandrillaris* (Figure 1a). The compounds β-amyrin, betulinic acid, rosmarinic acid, and ursolic acid showcased the highest cidal activity with only 66.52%, 62.15%, 62.17%, and 22.59% viable amoeba remaining, respectively. However, only three plant-based compounds showed significant amoebicidal activity against *N. fowleri* (Figure 1b). The three compounds, ursolic acid, betulinic acid, and betulin exhibited significant cidal activity with only 49.30%, 28.19%, and 14.82% viable amoeba remaining, respectively (Figure 1b).

Furthermore, the compounds showing significant amoebicidal activity were tested at different concentrations to determine their MIC_50_ (Table 1). The compounds showing significant amoebicidal properties against *B. mandrillaris* were tested at concentrations of 50 µg/mL, 100 µg/mL, 150 µg/mL, and 200 µg/mL (Table 1a). It was found that oleanolic acid, betulinic acid, β-amyrin, betulin, vanillic acid, rosmarinic acid, ursolic acid, and methy-β-orcinolcarboxylate inhibited 50% of *B. mandrillaris* growth at concentrations of: 189.6 µg/mL, 88.33 µg/mL, 112 µg/mL, 80.34 µg/mL, 132 µg/mL, 156.2 µg/mL, 131.3 µg/mL, and 139 µg/mL, respectively (Table 1a). Whereas betulinic acid, betulin, and ursolic acid inhibit 50% of *N. fowleri* growth at concentrations of 34.39 µg/mL, 77 µg/mL, and 74.67 µg/mL, respectively (Table 1b). 

### 3.2. The Plant-Based Compounds Exhibited Minimal Cytotoxic Activity against Human Cell Lines

**Figure 2:**
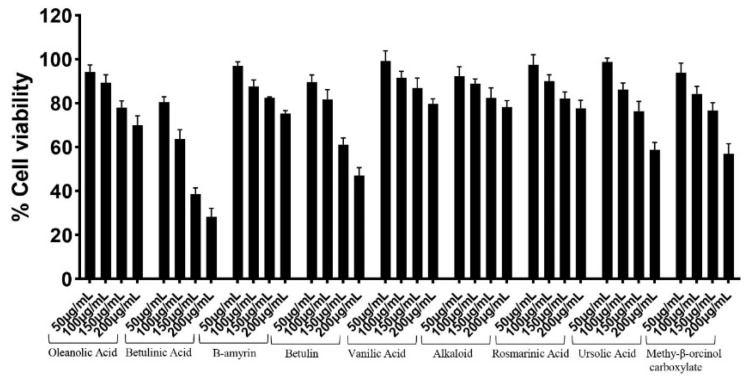
The plant-based compounds exhibited minimal cytotoxic activity against human cell lines. Confluent monolayers of HeLa cells were challenged with 50 µg/mL, 100 µg/mL, 150 µg/mL, and 200 µg/mL of the plant-based compounds. All compounds were non-cytotoxic at a concentration of 100 µg/mL except betulinic acid, as it exhibited weak cytotoxicity. Cell cytotoxicity below 20% is considered non-cytotoxic, while cell cytotoxicity between 20% to 40% is considered to have weak cytotoxicity. The data are illustrative of several independent experiments and presented as the mean ± standard error.

Lactate dehydrogenase assays were conducted to measure the toxicity of the plant-based compounds toward human cells. Concentrations of 50 µg/mL, 100 µg/mL, 150 µg/mL, and 200 µg/mL of the test compounds were tested against HeLa cells (Figure 2). It was found that at the working concentration of 100 µg/mL, eight of the nine compounds: oleanolic acid, β-amyrin, betulin, vvanillic acid, alkaloid, rosmarinic acid, ursolic acid, and methy-β-orcinolcarboxylate proved to be non-toxic; as cell cytotoxicity was approximately 20% and less. Of note is the compound betulinic acid, as it exhibited weak cytotoxic activity at 37%.

### 3.3. The Maximal Non-Toxic Dose and 50% Effective Concentration of Plant-Based Compounds against HeLa Cells Were Determined 

The maximal non-toxic dose (MNTD) and the 50% effective concentration (EC_50_) of the plant-derived compounds against HeLa cells were determined through the conduction of MTT assays. It was found that upon the addition of 334.4 µg/mL, 122,4 µg/mL, 432.7 µg/mL, 190.9 µg/mL, 440.4 µg/mL, 768.8 µg/mL, 449.9 µg/mL, 235.4 µg/mL and 241 µg/mL of oleanolic acid, betulinic acid, β-amyrin, betulin, vanillic acid, alkaloid, rosmarinic acid, ursolic acid, and methy-β-orcinolcarboxylate, respectively, were the compounds showing 50% effect against HeLa cells (Table 2). Additionally, the maximum non-toxic dose of oleanolic acid, betulinic acid, β-amyrin, betulin, vanillic acid, alkaloid, rosmarinic acid, ursolic acid, and methy-β-orcinolcarboxylate were found to be: 87.01 µg/mL, 36.98 µg/mL, 93.41 µg/mL, 63.37 µg/mL, 123.8 µg/mL, 77.46 µg/mL, 101.6 µg/mL, 93.05 µg/mL, and 81.89 µg/mL, respectively (Table 2).

### 3.4. The Plant-Based Compounds Reduced Amoebae-Mediated Host Cell Death

**Figure 3:**
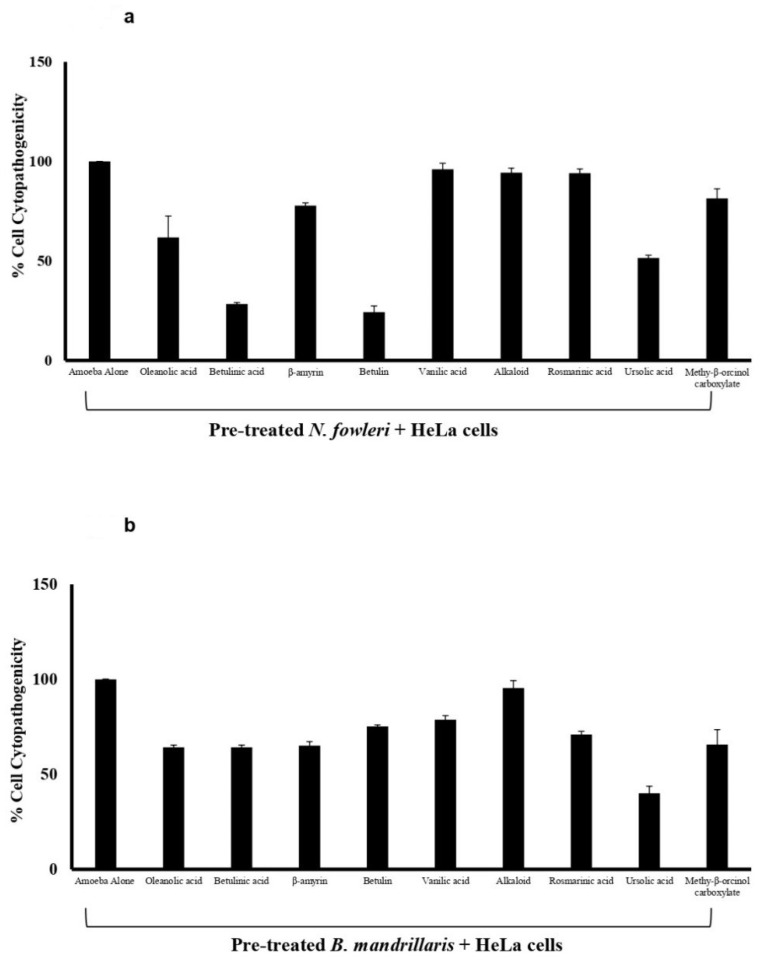
The plant-based compounds reduced amoebae-mediated host cell death. (**a**) The compounds reduced *B. mandrillaris* mediated cytotoxicity against human cells and (**b**) against *N. fowleri* mediated cytotoxicity against human cells. In short, 2 × 10^5^ amoebae were incubated with 100 µg/mL of the compounds for 2 h. After the 2-h incubation period was complete, the pre-treated amoebae were transferred to the HeLa cells and incubated overnight. Overall, the drugs are capable of inhibiting amoeba-meditated host cytotoxicity when compared to the amoeba alone. The data is illustrative of several independent experiments and presented as the mean ± standard error. The Y-axis error bars are indicative of standard error of the data depicted in the graphs. * corresponds to *p* < 0.05.

To evaluate the effect of the plant-based compounds on amoebae-mediated host cell death, cytopathogenicity assays were carried out. *B. mandrillaris* and *N. fowleri* were pre-treated with the test compounds before being introduced to the HeLa cell monolayer. The results revealed that upon the treatment of *B. mandrillaris* and *N. fowleri,* amoebae-mediated host cell death was reduced, compounds exhibiting significant amoebicidal activities, showed reduced amoeba-mediated host cell cytotoxicity (Figure 3).

**Figure 4:**
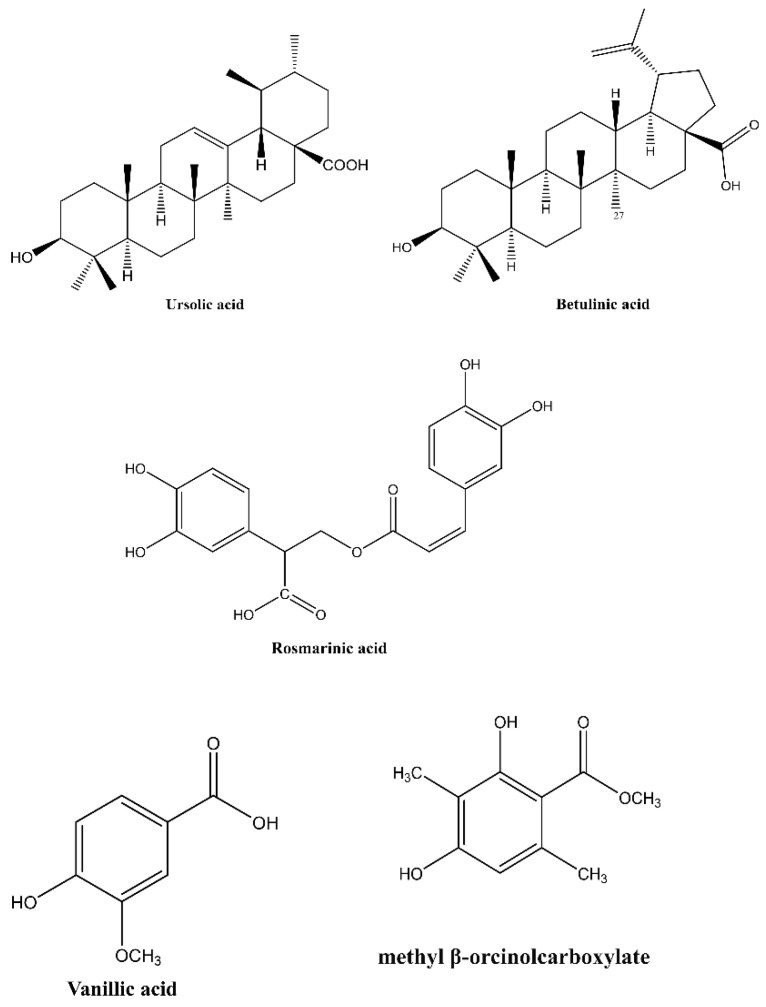
The molecular structure of active compounds.

Upon treating *B. mandrillaris* with the plant-derived compounds: oleanolic acid, betulinic acid, β-amyrin, betulin, vanillic acid, rosmarinic acid, and ursolic acid, the amoeba mediated host cell death was reduced from 100% to 64%, 64%, 65%, 75%, 79%, 71%, and 40%, respectively (Figure 3a). Additionally, upon the treatment of *N. fowleri* with the plant-derived compounds, betulinic acid, betulin, and ursolic acid reduced amoeba-mediated host cell death from 100% to 28%, 24%, and 51%, respectively (Figure 3b). The structure of active compounds is shown in Figure 4.

## 4. Discussion

*Balamuthia mandrillaris* and *Naegleria fowleri* are two highly fatal, protozoan pathogens distributed widely in the environment [3,26,27,28]. Furthermore, although these parasites are distributed globally, they favor warmer temperatures; hence, global warming is of concern [2,3]. Each parasite is responsible for a fatal central nervous system infection, to which, no effective treatment is currently present [1,2,18,29]. Unfortunately, current drugs possess a wide range of toxicities, as they are needed to be administered in high doses to be able to traverse the highly selective blood–brain barrier, as is the case with Amphotericin B [16,17]. For this reason, it is necessary to develop new treatments for these amoebae.

The use of medicinal plants to aid in treatment can be dated back to the ancient Greeks [19]. In this study, nine secondary metabolites from *Rinorea yaundensis* and *Salvia triloba* were tested against *N. fowleri* and *B. mandrillaris.* Oleanolic acid, betulinic acid, β-amyrin, betulin, vanillic acid, alkaloid, rosmarinic acid, ursolic acid, and methy-β-orcinolcarboxylate were tested for the anti-amoebic activities against the two amoebae. Furthermore, their cytopathic effects against human cell lines were also determined; the cytotoxicity, MNTD, and EC_50_ of the plant-derived compounds were determined against HeLa cells. Additionally, the amoeba-mediated host cell death was determined through the conduction of cytopathogenicity assays.

According to the results obtained, three of the nine test compounds exhibited significant amoebicidal activity against *N. fowleri*. The compounds: ursolic acid, betulinic acid, and betulin reduced amoeba viability, the greatest reduction was exhibited by betulin, where amoeba viability was reduced to 14.82%. Additionally, the compounds were also found to reduce *N. fowleri* mediated host cell death. Moreover, eight of the nine compounds tested: oleanolic acid, betulinic acid, β-amyrin, betulin, vanillic acid, rosmarinic acid, ursolic acid, and methy-β-orcinolcarboxylate showed significant anti-amoebic properties against *B. mandrillaris.* Of note are β-amyrin, betulinic acid, rosmarinic acid, and ursolic acid as they exhibited a significant reduction of amoeba viability. Additionally, the compounds oleanolic acid, betulinic acid, β-amyrin, betulin, vanillic acid, rosmarinic acid, and ursolic acid reduced amoebae-mediated host cell death. Although the exact mechanism of action is not known against *B. mandrillaris* and *N. fowleri*, compounds such as ursolic acid, betulinic acid, vanillic acid, and other plant-derived compounds have been found to induce apoptosis in cells [30,31]. Betulinic acid has been found to induce apoptosis by altering the mitochondrial function of tumor cells. Additionally, ursolic acid was found to induce apoptosis in *Acanthamoeba* by lowering the mitochondrial membrane potential and decreasing the ATP levels produced [30,31]. Additionally, the plant-derived compounds were tested against *Acanthamoeba* in a recent study; the compounds were found to exhibit significant amoebicidal activity against *Acanthamoeba* [24]. However, the mechanism of action of these compounds against *B. mandrillaris* and *N. fowleri* should be determined in future studies. Furthermore, as different concentrations of the plant-derived compounds were tested to determine the MIC_50_ values, only 34.39 µg/mL of betulinic acid is needed to inhibit 50% of *N. fowleri* growth, of the remaining compounds, betulinic acid showed the lowest concentration needed. Notably, previous studies showed that the sensitivities of the antifungal drugs (MIC_50_) were: amphotericin B (0.05–0.5 µg/mL), ketoconazole (0.125 µg/mL), fluconazole (0.5–2.0 mg/mL), and itraconazole (10 mg/mL) (*p* < 0.05) [32]. In another study, the MIC_100_ of amphotericin B, miltefosine, and chlorpromazine, were 0.78, 25, and 12.5 μg/mL, respectively [33]. For *B. mandrillaris*, amphotericin B, ciclopirox olamine, miltefosine, natamycin, paromomycin, pentamidine isethionate, protriptyline, spiramycin, sulconazole, and telithromycin had limited activity with amoebicidal levels of >135–500 μM [34]. However, diminazene aceturate (Berenil(^®^)) was amoebicidal at 7.8 μM and 31.3–61.5 μM for trophozoites and cysts [34]. Furthermore, in our study against *B. mandrillaris,* betulin was found to have the lowest MIC_50_ value, with only 80.34 µg/mL of the compound to inhibit 50% of *B. mandrillaris*.

Moreover, assays to determine the cytotoxic activities of the compounds against human cells were conducted. It was found that all compounds except betulinic acid depicted minimal cytotoxicity. Betulinic acid was found to exhibit 37% cytotoxicity against HeLa cells, thus, it depicts some toxic effects. Furthermore, the MNTD and EC_50_ of the compounds against the cells was determined. Betulinic acid exhibited the lowest EC_50_ value, where 122.4 µg/mL of the compound showed a 50% effectiveness against the human cells. Additionally, betulinic acid also exhibited the lowest MNTD value where 36.98 µg/mL of the compound is the maximum dose not toxic to the cells. Hence, it can be concluded that out of the nine compounds, betulinic acid is the most toxic to human cells.

When comparing antiamoebic activities with mammalian cell cytotoxicity for active compounds observed in this study, it was found that oleanolic acid, betulinic acid, β-amyrin, betulin, vanillic acid, rosmarinic acid, ursolic acid, and methy-β-orcinolcarboxylate inhibited 50% of *B. mandrillaris* at concentrations of: 189.6 µg/mL, 88.33 µg/mL, 112 µg/mL, 80.34 µg/mL, 132 µg/mL, 156.2 µg/mL, 131.3 µg/mL, and 139 µg/mL respectively. Whereas betulinic acid, betulin, and ursolic acid inhibit 50% of *N. fowleri* at concentrations of 34.39 µg/mL, 77 µg/mL, and 74.67 µg/mL, respectively. In comparison, oleanolic acid, betulinic acid, β-amyrin, betulin, vanillic acid, rosmarinic acid, ursolic acid, and methy-β-orcinolcarboxylate inhibited 50% of mammalian cells at 334.4 µg/mL, 122,4 µg/mL, 432.7 µg/mL, 190.9 µg/mL, 440.4 µg/mL, 449.9 µg/mL, 235.4 µg/mL and 241 µg/mL, respectively. These findings have identified several compounds that inhibit pathogenic amoebae without affecting human cells.

Unfortunately, current treatments against *B. mandrillaris* and *N. fowleri* have shown to be toxic, and capable of damaging tissues; hence, the plant-derived compounds are of particular importance as they exhibit minimal-cytotoxic activity and possess potent activity against the amoebae.

The compounds tested in this study have been previously noted for their various health benefits, for example, ursolic acid is a triterpene compound believed to have various health benefits such as anti-inflammatory, anti-carcinogenic, antioxidant, and anti-apoptotic effects [35]. Additionally, it is believed to reduce the expression of markers of cardiac damage in the heart, decrease inflammation and increase antioxidants in the brain as well as reduce apoptotic signals. Moreover, ursolic acid possesses antimicrobial activities, including anti-protozoal activities against *Plasmodium falciparum* [36]. Betulinic acid also possesses a wide range of pharmaceutical properties such as antitumor, antiviral, anti-inflammatory, and anti-diabetic properties, while Betulin possesses anticancer effects [37,38].

Although these compounds possess various pharmaceutical properties and show antiamoebic properties, further studies should be done. Initially, further testing against amoebae should be conducted such as encystation and excystation assays to understand the effect of the compounds against the amoebae cyst. Next, the mechanism of action against the amoebae should be determined using electron microscopic studies. Further testing can be accomplished in vivo using infected animal models such as mice. Moreover, the necessary mode of administration should be established. The amoebae are found in the brain; hence, the drugs should be able to bypass the highly selective blood–brain barrier without exhibiting toxic activity. Finally, the pharmacodynamic, pharmacokinetics, and efficacy of the effective compounds should be determined to establish the translational value of these findings.

## 5. Conclusions

Here we extracted and isolated plant-based natural compounds from two medicinal plants i.e., *R. yaundensis* and *S. triloba* and then tested them against *N. fowleri* and *B. mandrillaris*. The compounds tested displayed significant amoebicidal activity. These compounds inhibited amoeba-mediated host cell toxicity. All the compounds showed negligible cytotoxicity against human cells tested. These outcomes suggest that plant-based natural drugs entities hold promise in the improved treatment of infections caused by *N. fowleri* and *B. mandrillaris* and could open several avenues for further research against other parasites, as well as their capabilities as disinfectants for use in household water storage tanks, which will determine the translational value of these very promising findings.

## Figures and Tables

**Table 1:** The plant-based compounds exhibited significant amoebicidal activity against *N. fowleri* and *B. mandrillaris* The minimum inhibitory concentration required to inhibit 50% of parasite growth (MIC_50_) was determined for the compounds showing significant effects against *N. fowleri* and *B. mandrillaris* (**a**) concentrations of 50 µg/mL, 100 µg/mL, 150 µg/mL, and 200 µg/mL of eight plant-based compounds were tested against *B. mandrillaris* (**b**) concentrations of 50 µg/mL, 100 µg/mL and 200 µg/mL of three plant-based compounds were tested against *N. fowleri*.

***Balamuthia mandrillaris***					
	50 µg/mL	100 µg/mL	150 µg/mL	200 µg/mL	MIC50	
*Balamuthia mandrillaris* viability	100				
Oleanolic acid	91 ± 3.8	88 ± 4.4	83 ± 0.8	41 ± 9.2	189.6
Betulinic acid	77 ± 5.4	48 ± 4.7	17 ± 2.8	10 ± 4.5	88.33
β-amyrin	96 ± 3.4	63 ± 5.0	18 ± 1.6	16 ± 6.3	112
Betulin	81 ± 6.3	37 ± 3.2	1.6 ± 2.3	0 ± 0	80.34
Vanillic acid	96 ± 1.3	61 ± 9.7	47 ± 3.5	25 ± 4.1	132
Rosmarinic acid	85 ± 6.7	66 ± 2.7	55 ± 3.6	38 ± 3.3	156.2
Ursolic acid	87 ± 0.3	81 ± 1.6	27 ± 0.5	27 ± 0.5	131.3
Methyl-β-orcinolcarboxylate	87 ± 8.0	88 ± 0.9	39 ± 2.1	29 ± 1.9	139
***Naegleria fowleri***					
	50 µg/mL		100 µg/mL	200 µg/mL	MIC50
*Naegleria fowleri* viability	100				
Betulinic acid	77 ± 5.4		48 ± 4.7	10 ± 4.5	88.33
Betulin	81 ± 6.3		37 ± 3.2	0 ± 0	80.34
Ursolic acid	87 ± 0.3		81 ± 1.6	27 ± 0.5	131.3

**Table 2:** The maximal non-toxic dose and 50% effective concentration of plant-based compounds against HeLa cells were determined. The maximum non-toxic dose and the EC_50_ of the plant-based compounds were determined through the conduction of MTT assay.

Compounds	EC_50_	MNTD
Oleanolic acid	334.4	87.01
Betulinic acid	122.5	36.98
Β-amyrin	432.7	93.41
Betulin	190.9	63.37
Vanillic acid	440.4	123.8
Alkaloid	768.7	77.46
Rosmarinic acid	449.9	101.6
Ursolic acid	235.4	93.05
Methy-β-orcinolcarboxylate	241	81.89

## Data Availability

The data presented in this study are available on request from the corresponding author.

## References

[1] Khan N.A., Muhammad J.S., Siddiqui R. (2021). Brain-eating amoebae: Is killing the parasite our only option to prevent death?. Expert Rev. Anti-Infect. Ther..

[2] Siddiqui R., Khamis M., Ibrahim T., Khan N.A. (2021). Brain-Eating Amoebae in the United Arab Emirates?. ACS Pharmacol. Transl. Sci..

[3] Bhosale N.K., Parija S.C. (2021). *Balamuthia mandrillaris*: An opportunistic, free-living ameba—An updated review. Trop. Parasitol..

[4] Visvesvara G.S., Moura H., Schuster F.L. (2007). Pathogenic and opportunistic free-living amoebae: *Acanthamoeba* spp., *Balamuthia mandrillaris*, *Naegleria fowleri*, and *Sappinia diploidea*. FEMS Immunol. Med. Microbiol..

[5] Mungroo M.R., Anwar A., Khan N.A., Siddiqui R. (2020). Gold-Conjugated Curcumin as a Novel Therapeutic Agent against Brain-Eating Amoebae. ACS Omega.

[6] Pana A., Vijayan V., Anilkumar A.C. Amebic Meningoencephalitis. https://www.ncbi.nlm.nih.gov/books/NBK430754/?report=classic.

[7] Maciver S.K., Piñero J.E., Lorenzo-Morales J. (2020). Is *Naegleria fowleri* an emerging parasite?. Trends Parasitol..

[8] Qvarnstrom Y., Visvesvara G.S., Sriram R., da Silva A.J. (2006). Multiplex real-time PCR assay for simultaneous detection of *Acanthamoeba* spp., *Balamuthia mandrillaris*, and *Naegleria fowleri*. J. Clin. Microbiol..

[9] Gharpure R., Bliton J., Goodman A., Ali I.K.M., Yoder J., Cope J.R. (2021). Epidemiology and clinical characteristics of primary amebic meningoencephalitis caused by *Naegleria fowleri*: A global review. Clin. Infect. Dis..

[10] Debnath A. (2021). Drug discovery for primary amebic meningoencephalitis: From screen to identification of leads. Expert Rev. Anti-Infect. Ther..

[11] Capewell L.G., Harris A.M., Yoder J.S., Cope J.R., Eddy B.A., Roy S.L., Visvesvara G.S., Fox L.M., Beach M.J. (2015). Diagnosis, clinical course, and treatment of primary amoebic meningoencephalitis in the United States, 1937–2013. J. Pediatric Infect. Dis. Soc..

[12] Rice C.A., Lares-Jiménez L.F., Lares-Villa F., Kyle D.E. (2020). In vitro screening of the open source MMV Malaria and Pathogen Boxes to discover novel compounds with activity against *Balamuthia mandrillaris*. Antimicrob. Agents Chemother..

[13] Debnath A., Tunac J.B., Galindo-Gómez S., Silva-Olivares A., Shibayama M., McKerrow J.H. (2012). Corifungin, a new drug lead against *Naegleria*, identified from a high-throughput screen. Antimicrob. Agents Chemother..

[14] Rice C.A., Colon B.L., Alp M., Göker H., Boykin D.W., Kyle D.E. (2015). Bis-benzimidazole hits against *Naegleria fowleri* discovered with new high-throughput screens. Antimicrob. Agents Chemother..

[15] Troth E.V., Kyle D.E. (2021). EdU incorporation to assess cell proliferation and drug susceptibility in *Naegleria fowleri*. Antimicrob. Agents Chemother..

[16] Rajendran K., Anwar A., Khan N.A., Siddiqui R. (2017). Brain-eating amoebae: Silver nanoparticle conjugation enhanced efficacy of anti-amoebic drugs against *Naegleria fowleri*. ACS Chem. Neurosci..

[17] Grace E., Asbill S., Virga K. (2015). *Naegleria fowleri*: Pathogenesis, diagnosis, and treatment options. Antimicrob. Agents Chemother..

[18] Mungroo M.R., Khan N.A., Siddiqui R. (2020). *Balamuthia mandrillaris*: Pathogenesis, diagnosis, and treatment. Expert Opin. Orphan Drugs.

[19] Sakkas H., Papadopoulou C. (2017). Antimicrobial activity of basil, oregano, and thyme essential oils. J. Microbiol. Biotechnol..

[20] Warowicka A., Nawrot R., Goździcka-Józefiak A. (2020). Antiviral activity of berberine. Arch. Virol..

[21] Thomford N.E., Senthebane D.A., Rowe A., Munro D., Seele P., Maroyi A., Dzobo K. (2018). Natural products for drug discovery in the 21st century: Innovations for novel drug discovery. Int. J. Mol. Sci..

[22] Atanasov A.G., Zotchev S.B., Dirsch V.M., Supuran C.T. (2021). Natural products in drug discovery: Advances and opportunities. Nat. Rev. Drug Discov..

[23] Manandhar S., Luitel S., Dahal R.K. (2019). In vitro antimicrobial activity of some medicinal plants against human pathogenic bacteria. J. Trop. Med..

[24] Siddiqui R., Akbar N., Khatoon B., Kawish M., Ali M.S., Shah M.R., Khan N.A. (2022). Novel Plant-Based Metabolites as Disinfectants against *Acanthamoeba castellanii*. Antibiotics.

[25] Akbar N., Khan N.A., Sagathevan K., Iqbal M., Tawab A., Siddiqui R. (2019). Gut bacteria of *Cuora amboinensis* (turtle) produce broad-spectrum antibacterial molecules. Sci. Rep..

[26] Güémez A., García E. (2021). Primary Amoebic Meningoencephalitis by *Naegleria fowleri*: Pathogenesis and Treatments. Biomolecules.

[27] Herman E.K., Greninger A., van der Giezen M., Ginger M.L., Ramirez-Macias I., Miller H.C., Morgan M.J., Tsaousis A.D., Velle K., Vargová R. (2021). Genomics and transcriptomics yields a system-level view of the biology of the pathogen *Naegleria fowleri*. BMC Biol..

[28] Safavi M., Mehrtash V., Habibi Z., Mohammadpour M., Mohammad T.H.A., Zaresharifi N., Shafizadeh M., Jafarzadeh B. (2021). Case Report: Encephalitis Caused by *Balamuthia mandrillaris* in a 3-Year-Old Iranian Girl. Am. J. Trop. Med. Hyg..

[29] Rice C.A., Troth E.V., Russell A., Kyle D.E. (2020). Discovery of anti-amoebic inhibitors from screening the MMV pandemic response box on *Balamuthia mandrillaris*, *Naegleria fowleri*, and *Acanthamoeba castellanii*. Pathogens.

[30] Sifaoui I., Rodríguez-Expósito R.L., Reyes-Batlle M., Rizo-Liendo A., Piñero J.E., Bazzocchi I.L., Lorenzo-Morales J., Jiménez I.A. (2019). Ursolic acid derivatives as potential agents against *Acanthamoeba* spp.. Pathogens.

[31] Mahboob T., Azlan A.M., Shipton F.N., Boonroumkaew P., Azman N.S.N., Sekaran S.D., Ithoi I., Tan T.C., Samudi C., Wiart C. (2017). Acanthamoebicidal activity of periglaucine A and betulinic acid from *Pericampylus glaucus* (Lam.) Merr. in vitro. Exp. Parasitol..

[32] Tiewcharoen S., Junnu V., Chinabut P. (2002). In vitro effect of antifungal drugs on pathogenic *Naegleria* spp.. Southeast Asian J. Trop. Med. Public Health.

[33] Kim J.H., Jung S.Y., Lee Y.J., Song K.J., Kwon D., Kim K., Park S., Im K.I., Shin H.J. (2008). Effect of therapeutic chemical agents in vitro and on experimental meningoencephalitis due to *Naegleria fowleri*. Antimicrob. Agents Chemother..

[34] Ahmad A.F., Heaselgrave W., Andrew P.W., Kilvington S. (2013). The in vitro efficacy of antimicrobial agents against the pathogenic free-living amoeba *Balamuthia mandrillaris*. J. Eukaryot. Microbiol..

[35] Seo D.Y., Lee S.R., Heo J.W., No M.H., Rhee B.D., Ko K.S., Kwak H.B., Han J. (2018). Ursolic acid in health and disease. Korean J. Physiol. Pharmacol..

[36] Do Nascimento P.G., Lemos T.L., Bizerra A., Arriaga Â., Ferreira D.A., Santiago G.M., Braz-Filho R., Costa J.G.M. (2014). Antibacterial and antioxidant activities of ursolic acid and derivatives. Molecules.

[37] Ríos J.L., Máñez S. (2018). New pharmacological opportunities for betulinic acid. Planta Med..

[38] Król S.K., Kiełbus M., Rivero-Müller A., Stepulak A. (2015). Comprehensive review on betulin as a potent anticancer agent. BioMed Res. Int..

